# Molecular phenotyping of domestic cat (*Felis catus*) testicular cells across postnatal development – A model for wild felids

**DOI:** 10.1016/j.therwi.2023.100031

**Published:** 2023-04-20

**Authors:** M. Bashawat, B.C. Braun, K. Müller, B.P. Hermann

**Affiliations:** a Department of Biology, Humboldt University of Berlin, Invalidenstr. 42, D-10115 Berlin, Germany; b Department of Reproduction Biology, Leibniz Institute for Zoo and Wildlife Research, Alfred-Kowalke-Str. 17, D-10315 Berlin, Germany; c Department of Neuroscience, Developmental and Regenerative Biology, The University of Texas at San Antonio, San Antonio, TX 78249, USA

**Keywords:** Feline, Spermatogenesis, Testis tissue, Testicular cells, Conservation

## Abstract

Molecular characterisation of testicular cells is a pivotal step towards a profound understanding of spermatogenesis and developing assisted reproductive techniques (ARTs) based on germline preservation. To enable the identification of testicular somatic and spermatogenic cell types in felids, we investigated the expression of five molecular markers at the protein level in testes from domestic cats (*Felis catus*) at different developmental phases (prepubertal, pubertal I and II, postpubertal I and II) classified by single-cell ploidy analysis. Our findings indicate a prominent co-labelling for two spermatogonial markers, UCHL1 and FOXO1, throughout postnatal testis development. Smaller subsets of UCHL1 or FOXO1 single-positive spermatogonia were also evident, with the FOXO1 single-positive spermatogonia predominantly observed in prepubertal testes. As expected, DDX4+ germ cells increased in numbers beginning in puberty, reaching a maximum at adulthood (post-pubertal phase), corresponding to the sequential appearance of labelled spermatogonia, spermatocytes and spermatids. Furthermore, we identified SOX9+ Sertoli cells and CYP17A1+ Leydig cells in all of the developmental groups. Importantly, testes of African lion (*Panthera leo*), Sumatran tiger (*Panthera tigris sumatrae*), Chinese leopard (*Panthera pardus japonesis*) and Sudan cheetah (*Acinonyx jubatus soemmeringii*) exhibited conserved labelling for UCHL1, FOXO1, DDX4, SOX9 and CYP17A1. The present study provides fundamental information about the identity of spermatogenic and somatic testicular cell types across felid development that will be useful for developing ART approaches to support endangered felid conservation.

## 1. Introduction

In 2022, the International Union for Conservation of Nature (IUCN) listed 18 feline species as either vulnerable or endangered due to the risk of extinction [1]. Therefore, it is imperative to develop strategies that will help to prevent the decline in genetic diversity and imminent loss of these species in the coming decades. Sperm cryopreservation and artificial insemination are essential assisted reproductive technologies (ARTs) employed to bank and disseminate valuable genetic resources and preserve the male germline in endangered species [2,3]. However, spermatozoa are differentiated haploid cells that are no longer able to replicate, so banked sperm are not a renewable resource [4]. Moreover, spermatogenesis only begins during pubertal development in mammals, and thus, prepubertal individuals cannot bank sperm. Therefore, additional tools are needed to preserve the germline, particularly in endangered feline species, through a means that does not require obtaining mature sperm.

As an alternative to the recovery of sperm, testicular tissue that contains less mature spermatogenic cells represents a resource with high potential for fertility restoration and species conservation [5]. Cells isolated from testicular tissue could be used directly for fertilisation or to generate fertilisation-competent gametes. For instance, round spermatid injection (ROSI), in which frozen-thawed haploid, and morphologically immature, round spermatids are injected into human oocytes, has led to live births in humans [6]. It is also possible to produce fertilisation-competent sperm from spermatogonia through auto-, allo- and xeno-transplantation into recipient testes [7,8]. Finally, fresh or frozen tissue fragments from the immature testes of mice [9], rats [10], non-human primates [11,12] and humans [13] have been used to generate haploid germ cells by grafting into host animals or exclusively *in vitro* using air-liquid interface culture approaches.

At its foundation, spermatogenesis relies on the proliferative activity of spermatogonia to sustain a high level of gametogenic output throughout the male reproductive lifespan. Spermatogonia represent all mitotic germ cells in the postnatal testis and comprise spermatogonial stem cells (SSCs, a subtype of undifferentiated germ cells capable of perpetual self-renewal), progenitor spermatogonia (a subtype of undifferentiated germ cells lacking perpetual self-renew capacity), and differentiating spermatogonia (which ultimately enter meiosis and transform into haploid cells, *i.e.*, finally, spermatozoa) [reviewed by [14]]. Supported by poorly defined contributions from testicular somatic cells (*e.g.*, Sertoli cells, peritubular myoid cells, Leydig cells), SSCs are the only spermatogenic cells that exhibit regenerative potential and this activity is present throughout postnatal development [15]. Therefore, SSC cryopreservation provides a renewable option to safeguard the gene pool of prepubertal individuals who are not yet producing haploid cells. Recently, we demonstrated successful cryopreservation of dissociated testicular cells of the domestic cat, cheetah, and Asiatic golden cat with post-thaw viability of roughly 45 % [16]. In order to determine which cells survive the cryopreservation/thaw process, it is essential to identify markers of feline testicular cells. Those markers are also needed to prove enrichment procedures for specific cell types, e. g. spermatogonia.

Unlike in mice or humans, characterisation of feline testicular cell types, and in particular, subtypes of spermatogonia, is still challenging since there is a paucity of antibodies raised against feline antigens. Indeed, the few studies where immunostaining of feline testicular cells has been performed relied on antibodies validated for cross-reactivity in various species, but not in felines [17–21]. Additionally, disparate tissue fixation, embedding, antigen retrieval and immunostaining protocols across research groups are hurdles that produce conflicting results that are difficult to reconcile.

In the present study, we sought to validate reagents that facilitate the identification of testicular somatic and spermatogenic cell types in felids to provide a resource for the research community investigating cat spermatogenesis. Here, results for five testicular cells markers in testes from domestic cats and four wild feline species demonstrate successful immunolocalization with either frozen or paraffin-embedded sections across testis development. Importantly, our results demonstrate substantial marker conservation across species, validating the use of the domestic cat as a model for engendered feline species.

## 2. Material and methods

### 2.1. Preparation of testis tissue

Testis tissue of domestic cats (*Felis catus*, n = 10) was obtained as biowaste from veterinary clinics in Berlin (Germany). Additionally, testes of one African lion (*Panthera leo*), one Sumatran tiger (*Panthera tigris sumatrae*) and one Chinese leopard (*Panthera pardus japonesis*), all from Ree Park Safari (Ebeltoft, Denmark; 56°15’46.1”N 10°44’17.0”E) and one Sudan cheetah (*Acinonyx jubatus soemmeringii*), obtained from Landau Zoo (Landau, Germany; 49°12’13.3”N 8°06’40.5”E), were used in the study. Testes of wild feline species were obtained following euthanasia due to health-related problems or population management reasons according to the European Association of Zoos and Aquaria (EAZA) culling statement (https://www.eaza.net). Testes were transported and stored at 4 °C for less than 24 h before processing.

Upon arrival in the laboratory, the epididymides were dissected and the presence of spermatozoa was examined by light microscopy after mincing the cauda epididymis and the proximal ductus deferens together in 1 mL culture medium M199 (Hepes modification, Sigma-Aldrich M7528). After weighing each testis, one part of the testicular parenchyma was snap-frozen in liquid nitrogen and stored at − 80 °C for ploidy-based analysis of developmental phase. The remainder of the testis tissue was prepared for immunostaining. For domestic cats, fragments from two different males were used for immunostaining at each developmental phase (determined retrospectively).

All experiments were approved by the internal committee for Ethics and Animal Welfare of Leibniz Institute for Zoo and Wildlife Research (IZW), Berlin, (Germany) (Assurance no. 2020-06-04).

### 2.2. Determination of developmental phases

Classification of testis samples by developmental phase was done according to criteria set by Braun et al. [22], including: testis weight, presence or absence of spermatozoa in the cauda epididymis, histological characterisation and the flow cytometry-based DNA content analysis (ploidy analysis). For histological analyses, H&E stained sections of testicular tissue were viewed under a microscope to determine the most advanced germ cell phase [22], whereas the ploidy analysis was performed as described earlier by Blottner and Jewgenow [23]. Briefly, frozen testicular parenchyma (0.05 g) was thawed and finely minced followed by agitation for 20 min at room temperature in 0.8 mL of 100 mM citric acid containing 0.5 % (v/v) Tween 20 to release nuclei. Next, the DNA content in isolated nuclei was stained with 5 μM 4′,6-Diamidino-2-phenylindole dihydrochloride (DAPI) for 10 min in the dark. Flow cytometric analysis was performed using a PAS III flow cytometer (Sysmex Deutschland GmbH, Germany) equipped with an UV LED and an appropriate filter set (excitation: 360 nm; emission: 420 nm). FlowMax software (Version 2.9, Sysmex Deutschland GmbH, Germany) was used to count nuclei and determine their ploidy (1C, 2C and 4C) including, haploid (spermatids and spermatozoa), diploid (spermatogonia, preleptotene and secondary spermatocytes and somatic cells) and tetraploid cells (all cells in the G2/M phase of the mitotic or meiotic cell cycles, mainly primary spermatocytes). For each developmental phase, two different animals (1 and 2) were utilised for the immunostaining experiments and testicular cell counting. Completion of spermatogenesis was determined by the presence of spermatozoa in the cauda epididymis. A decrease in ratio 2C/4C indicates an increase in cell division (mitosis and first part of meiosis); an increase in ratio 1C/2C indicates an increase in meiotic activity, and thus, spermatid production.

### 2.3. Testis tissue immunostaining

Testis tissue from each individual animal (domestic cat or wild felid) was split and used for 1) cryosectioning and immunostaining according to Hermann et al. [15] and 2) paraffin-embedding and immunostaining according to Braun et al. [24]. Briefly, for cryosectioning, testis tissue fragments were fixed with 4 % (w/v) paraformaldehyde (PFA) for 24 h at 4 °C. After fixation, the fragments were washed three times with Dulbecco’s Phosphate Buffered Salt Solution (DPBS, Sigma-Aldrich D8537) and cryoprotected through sequential immersion in sucrose solutions of increasing concentration [10 %, 20 % and 30 % (w/v)]. Finally, the testis tissue fragments were embedded in an OCT medium (Sakura Finetek Europe B.V), frozen on dry ice and stored at − 80 °C. From each cryo-embedded testis tissue fragment, 5 μm serial sections were cut at − 20 °C and placed on positively-charged slides (Superfrost Plus, Fisher Scientific, USA). Slides were stored at − 80 °C prior to use. Sections were blocked with blocking buffer (DPBS containing 10 % (v/v) FBS, 5 % (w/v) BSA and 0.03 % Triton X-100 (v/v)) for 2 h at 4 °C and incubated overnight at 4 °C with the primary antibody diluted in blocking buffer (see list of antibodies and working dilutions in Table 1). Sections were then washed with DPBS containing 0.1 % Tween-20 (v/v). The appropriate secondary antibody [Table 2, all from Thermo Fisher Scientific (Illinois, USA)] diluted in blocking buffer together with DAPI were added for 45 min at room temperature followed by two successive washing steps with DPBS containing 0.1 % Tween-20 (v/v). Primary antibodies were omitted as negative controls (for exemplary control images, see Suppl. Fig. 4). Finally, sections were mounted with Prolong Gold antifade mounting medium (Thermo Fischer Scientific, USA), coverslipped and imaged using an AxioImager M1 microscope equipped with Axiocam MRm camera and the Zen Blue software (Version 10.0.19042) (all from Carl Zeiss Microscopy GmbH, Germany).

For paraffin-embedding, tissue was fixed with Bouińs fixative (24 h) before dehydration (increasing ethanol-series and Xylol) followed by embedding in Paraplast Plus Wax (Leica). Section (3 μm) of paraffin-embedded testis samples were mounted on charged microscope slides (Superfrost Plus, Thermo Scientific, Braunschweig, Germany), deparaffinized in Roti-Histol (Carl Roth GmbH, Karlsruhe, Germany), and rehydrated in a graded ethanol series. Sections were then washed with PBS and subjected to heat-induced antigen retrieval using citrate buffer pH 6.0 for 15 min, cooled for 20 min, washed, blocked of endogenous peroxidases with 3 % H_2_O_2_ in methanol for 10 min, washed, and blocked again with 5 % BSA in PBS for 1 h; all at room temperature. Incubation with primary antibodies (see Table 1) diluted in 1 % BSA in PBS was overnight at 4 °C whereas primary antibodies were omitted for negative controls (for exemplary control images, see Suppl. Fig. 5). After washing, sections were then incubated with HRP-conjugated secondary antibodies [BIOZOL Diagnostics Vertrieb GmbH (Eching, Germany)] and washed again. Immunoperoxidase localisation was detected with diaminobenzidine substrate chromogen solution (Dako Deutschland GmbH, Hamburg, Germany) and sections were counterstained with hematoxylin, dehydrated in a graded ethanol series before coverslipping with ROTI^®^Histokitt mounting medium (Carl Roth GmbH, Karlsruhe, Germany). Slides were analysed with an Olympus IX81 microscope combined with a DP72 camera and the cellSens Dimension Software (Version 1.18) (all from Olympus Deutschland GmbH, Hamburg, Germany).

### 2.4. Quantification of testicular cell types

The ImageJ software (Version 1.53c, NIH, Bethesda, MD; http://imagej.nih.gov/ij/) was used for counting domestic cat testicular cells, positive for SOX9, UCHL1 and DDX4 in round seminiferous tubule cross-sections. Numbers of labelled testicular cells were counted in 24 seminiferous tubule cross-sections from each animal at each developmental phase. The average numbers and the ratios of SOX9, UCHL1 and DDX4 positive cells in relation to DAPI positive cells per seminiferous tubule cross-section were calculated for each developmental phase.

### 2.5. Statistical analysis

Statistical analysis and graphical presentation were performed using R software (R Language and Environment for Statistical Computing and Graphics, version 4.1.2; Vienna, Austria). Kruskal-Wallis rank sum test was utilised to define differences between developmental phases (numbers and percentages of SOX9, UCHL1 and DDX4 positive testicular cells). If a significant effect was indicated by p ≤ 0.05, pairwise comparisons were performed using Wilcoxon signed rank test with correction for multiple testing (Benjamini-Hochberg method). The Shapiro–Wilk test of normality was used to determine if the variables followed a normal distribution.

## 3. Results

### 3.1. Determination of developmental phases of domestic cats and wild felids

Testis developmental phase was determined by calculating the ratios between haploid, diploid and tetraploid cells (1C/2C, 1C/4C and 2C/4C; Table 3 – domestic cat and Supplementary Table 1 – wild felids). Classification of developmental phases revealed five phases, namely, prepubertal, pubertal I, pubertal II, postpubertal I and postpubertal II.

### 3.2. SOX9 specifically marks domestic cat Sertoli cells

**Fig. 1.**
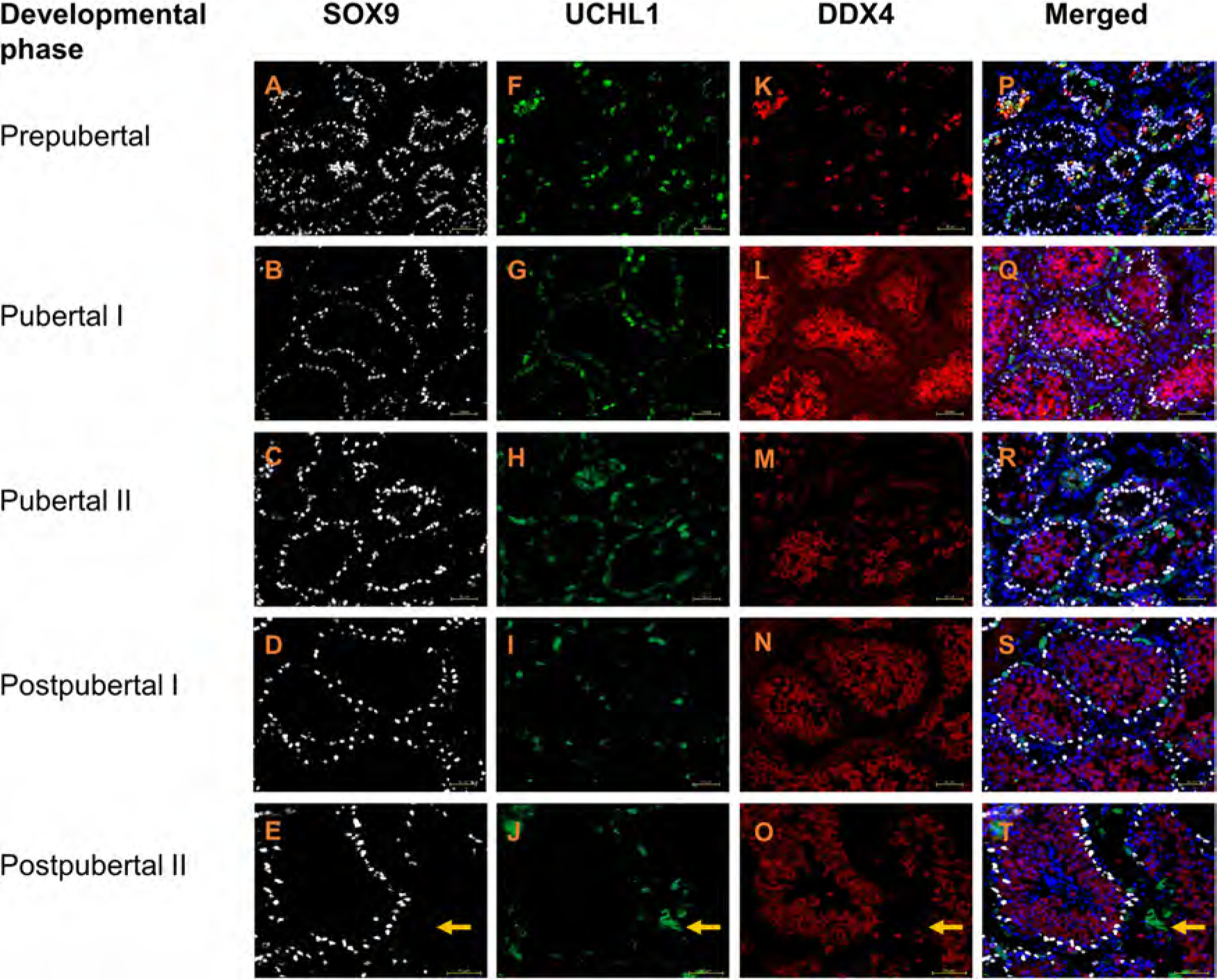
Testis tissue of domestic cats from five developmental phases stained with SOX9 (white), UCHL1 (green) and DDX4 (red) antibodies. Nuclei (blue) stained with DAPI. Scale bar: 50μm. Note: In postpubertal II phase nonspecific labelling was observed in the UCHL1 fluorescent channel (Arrow). The corresponding secondary antibody in the absence of UCHL1 antibody showed also unspecific interstitial labelling in testes of postpubertal II individuals (see Suppl. Fig. 4).

Nuclear labelling with the anti-SOX9 antibody was evident in a subset of seminiferous tubular cells. The SOX9+ cells were considered likely Sertoli cells based on their localisation and nuclear shape in both cryo-embedded and paraffin-embedded cross-sections, as well as according to the morphological appearance in the paraffin-embedded cross-sections (Fig. 1 A–E, Suppl. Fig. 1 A–F). We did not observe overlapping staining between SOX9+ nuclei and germ cell markers (DDX4 or UCHL1) in the cryo-embedded samples (Fig. 1 P–T, Suppl. Fig. 2) and did not see SOX9 labelling of germ cells or other testicular somatic cell types in the paraffin-embedded samples (Fig. 1 A–E, Suppl. Fig. 1 A–F).

### 3.3. CYP17A1 antibody specifically labels Leydig cells of domestic cat

**Fig. 2.**
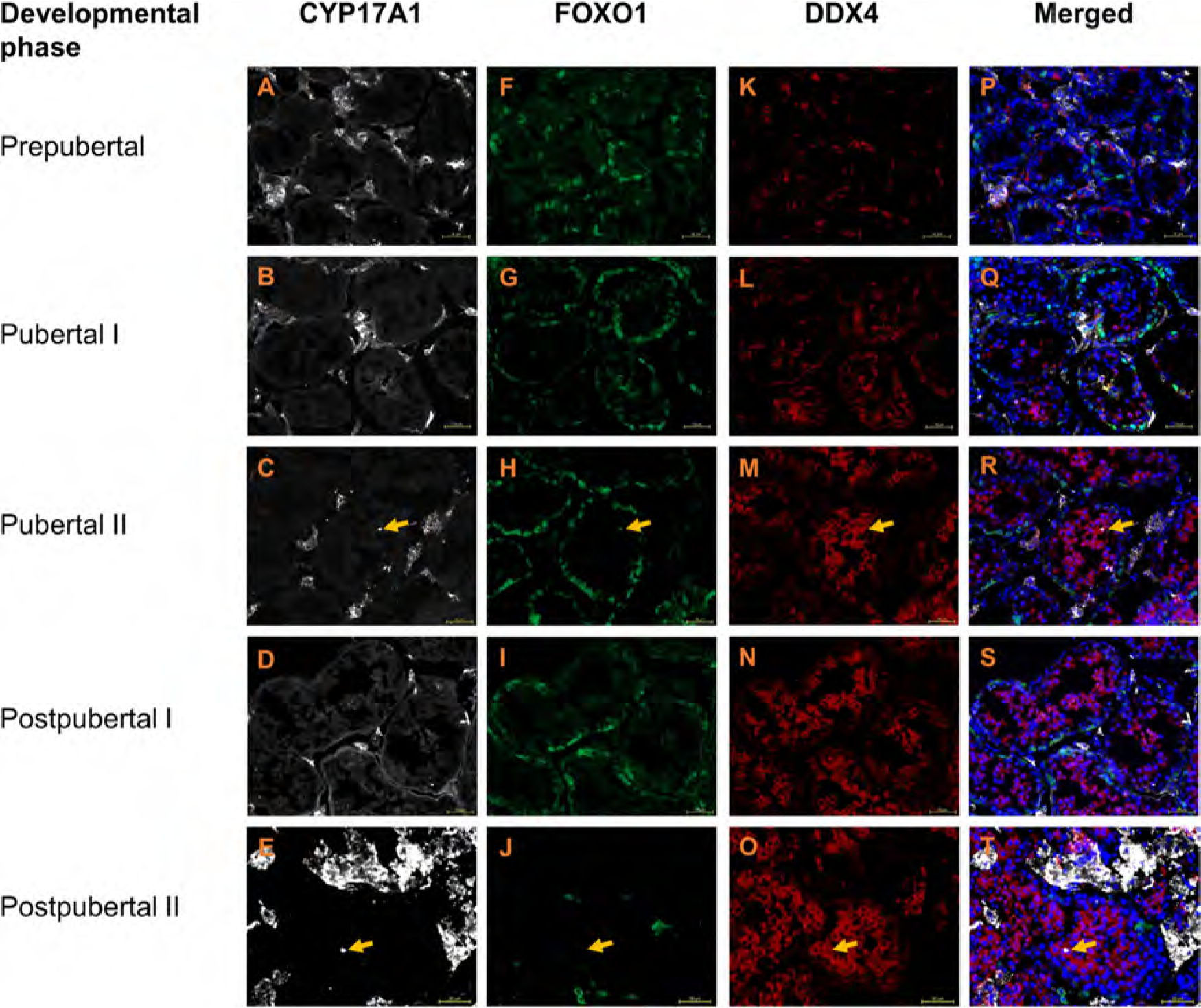
Testis tissue of domestic cats from five developmental phases stained with CYP17A1 (white), FOXO1 (green) and DDX4 (red) antibodies. Nuclei (blue) stained with DAPI. Scale bar: 50 μm. The dot-like labelling in C, H and F (arrow) is most likely related to precipitated antibody molecules.

The immunostaining experiments with CYP17A1 antibody revealed the expected cytoplasmic staining of interstitial cells at all developmental phases representing likely Leydig cells (Fig. 2 A–E). No tubular labelling with CYP17A1 antibody was observed.

### 3.4. UCHL1 and FOXO1 specifically mark spermatogonia of domestic cat

**Fig. 3.**
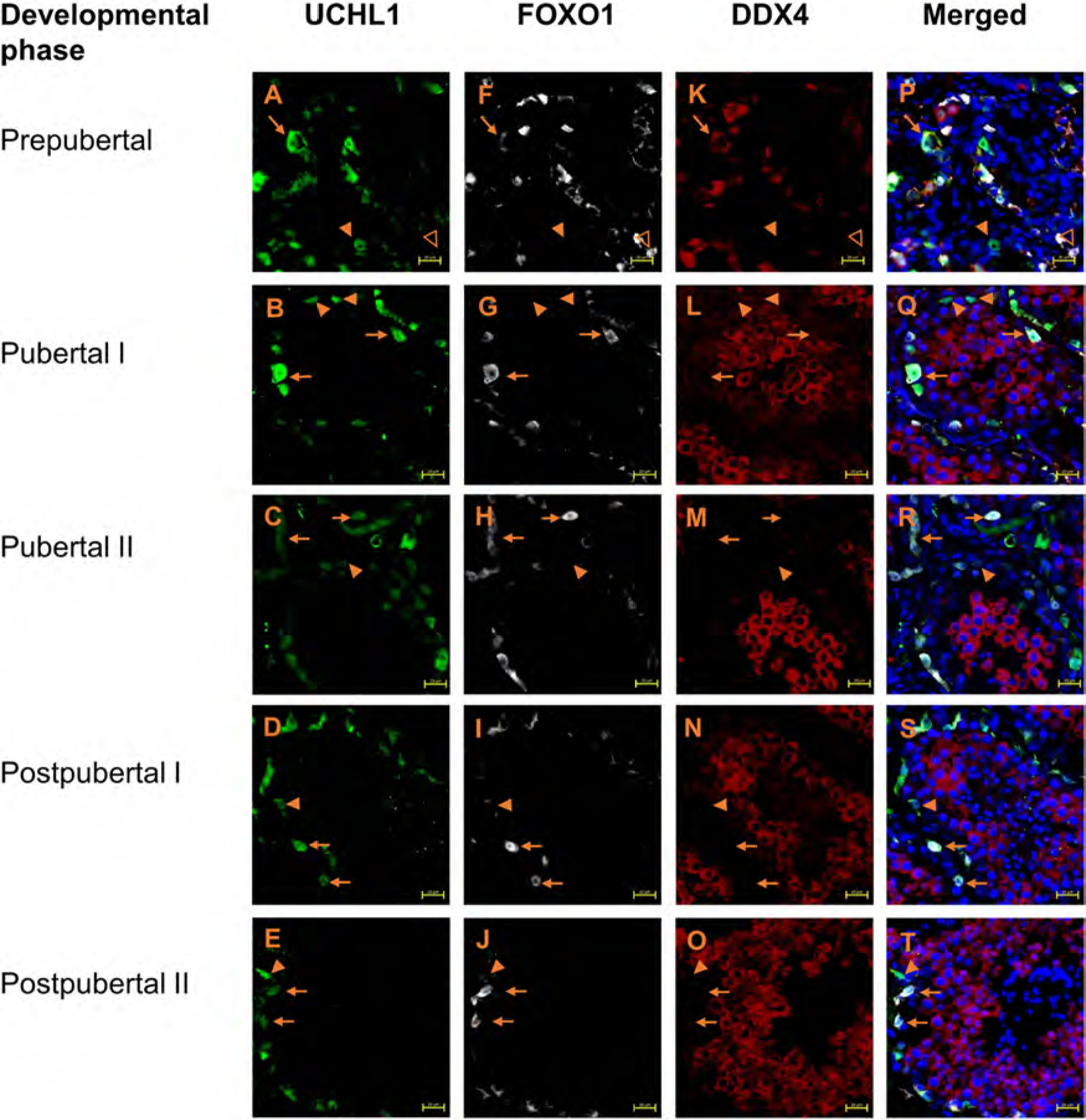
Examples of the staining patterns of UCHL1 (green), FOXO1 (white) and DDX4 (red) antibodies in spermatogonia of domestic cats at different developmental phases. Nuclei (blue) stained with DAPI. Scale bar: 20 μm. The arrow refers to the spermatogonia positive for UCHL1 and FOXO1. The solid triangle refers to the spermatogonia positive for UCHL1 and negative for FOXO1. The empty triangle refers to the spermatogonia negative for UCHL1 and positive for FOXO1.

In the domestic cat testis, UCHL1+ and FOXO1+ cells were evident at all developmental phases (Figs. 1 and 2, F–J, respectively, Fig. 3). Testicular cells positive for UCHL1 antibody were interspersed between SOX9+ Sertoli cell nuclei (Suppl. Fig. 2), and both UCHL1+ and FOXO1+ positive cells were localised to the basement membrane or seminiferous tubules (Figs. 1 and 2, F–J, respectively, Fig. 3 A–J, P–T). The morphological appearance of UCHL1+ and FOXO1+ cells was similar with round or oval flattened large nuclei. The labelling pattern for UCHL1 was identical in paraffin-embedded cross sections with chromogenic detection (Suppl. Fig. 1 G–L).

Since both UCHL1 and FOXO1 should be expressed by spermatogonia, triple-immunostaining for UCHL1, FOXO1 and the pan germ cell marker DDX4 was performed (Fig. 3). At all developmental phases, the majority of spermatogonia were double-positive for UCHL1 and FOXO1 (Fig. 3 P–T). Additionally, smaller subsets of UCHL1 or FOXO1 single-positive spermatogonia were also evident (Fig. 3 P–T), with FOXO1 single-positive spermatogonia being exclusive to prepubertal testes (Fig. 3 P). In prepubertal and pubertal testes, cytoplasmic FOXO1 labelling was observed, while in postpubertal phases, both cytoplasmic and nuclear FOXO1 were present.

Notably, unexpected UCHL1 labelling was evident among interstitial cells in the cryo-embedded samples from both postpubertal II individuals (Fig. 1 J). This peculiarity was also observed in paraffin-embedded sections from only one individual domestic cat (Suppl. Fig. 1 K. Negative controls labelled with only the secondary antibodies in the cryo-embedded samples showed similar fluorescence signal in the interstitial compartments of postpubertal domestic cat (Suppl. Fig. 4 F and G) suggesting autofluorescence. Respective controls with paraffin-embedded samples were unstained (Suppl. Fig. 5), which would be consistent with a specific interstitial UCHL1 labelling in the corresponding sample.

### 3.5. DDX4 marks a wide range of domestic cat spermatogenic cells

As expected, DDX4 labelling was present in domestic cat spermatogenic cells at all developmental phases and absent from Sertoli cells, Leydig cells and other testicular somatic cells. DDX4 co-labelled the cytoplasm of cells also positive for spermatogonial markers UCHL1 and/or FOXO1 in prepubertal testes (Fig. 1 P, 2 P and 3 P, Suppl. Fig. 2). In pubertal and postpubertal phases, the cytoplasm of the adluminal spermatogenic cells (spermatocytes and round spermatids) were also DDX4+. However, the intensity of DDX4 labelling was generally lower in spermatogonia on the basement membrane than in adluminal germ cell types and was not evident in elongated spermatids.

### 3.6. Quantification of SOX9, UCHL1 and DDX4 positive cells

**Fig. 4.**
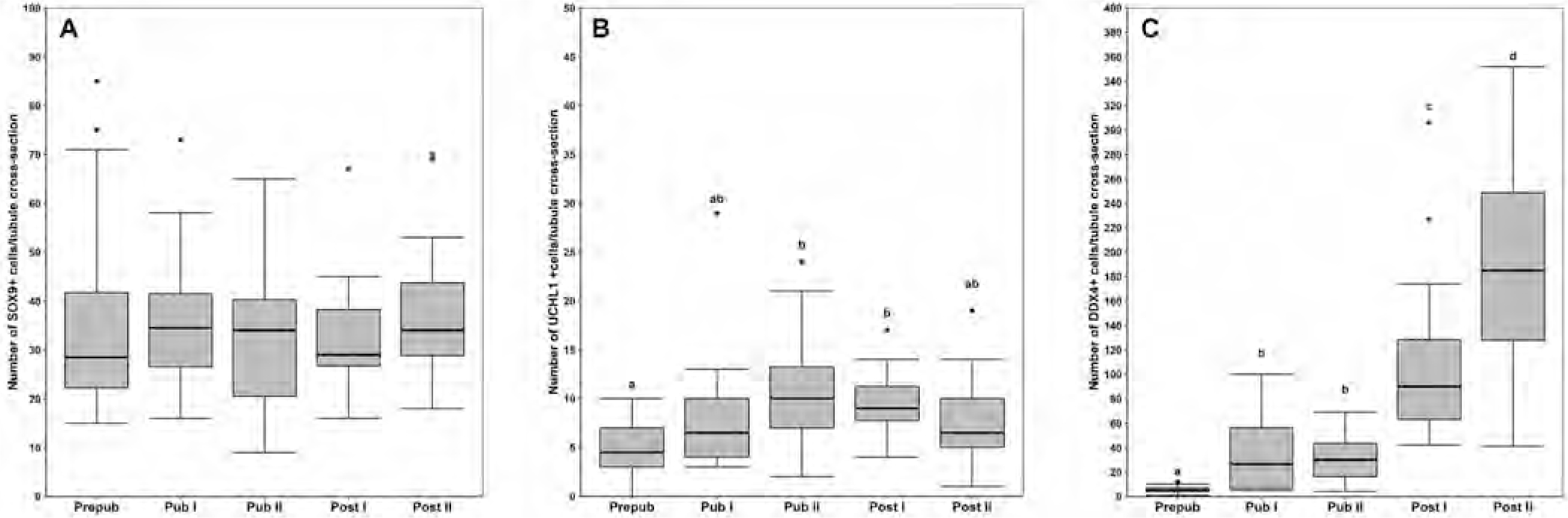
Changes between domestic cat developmental phases in the number of SOX9 (A), UCHL1 (B) and DDX4 (C) positive cells per seminiferous tubule cross-section. Data of 24 different seminiferous tubule cross-sections from two animals per developmental phase are displayed by means of box plots. Medians and the 25 and 75th percentiles are shown. Outliers are denoted by circles. Significant differences between developmental phases are indicated by different letters (P < 0.05).

**Fig. 5.**
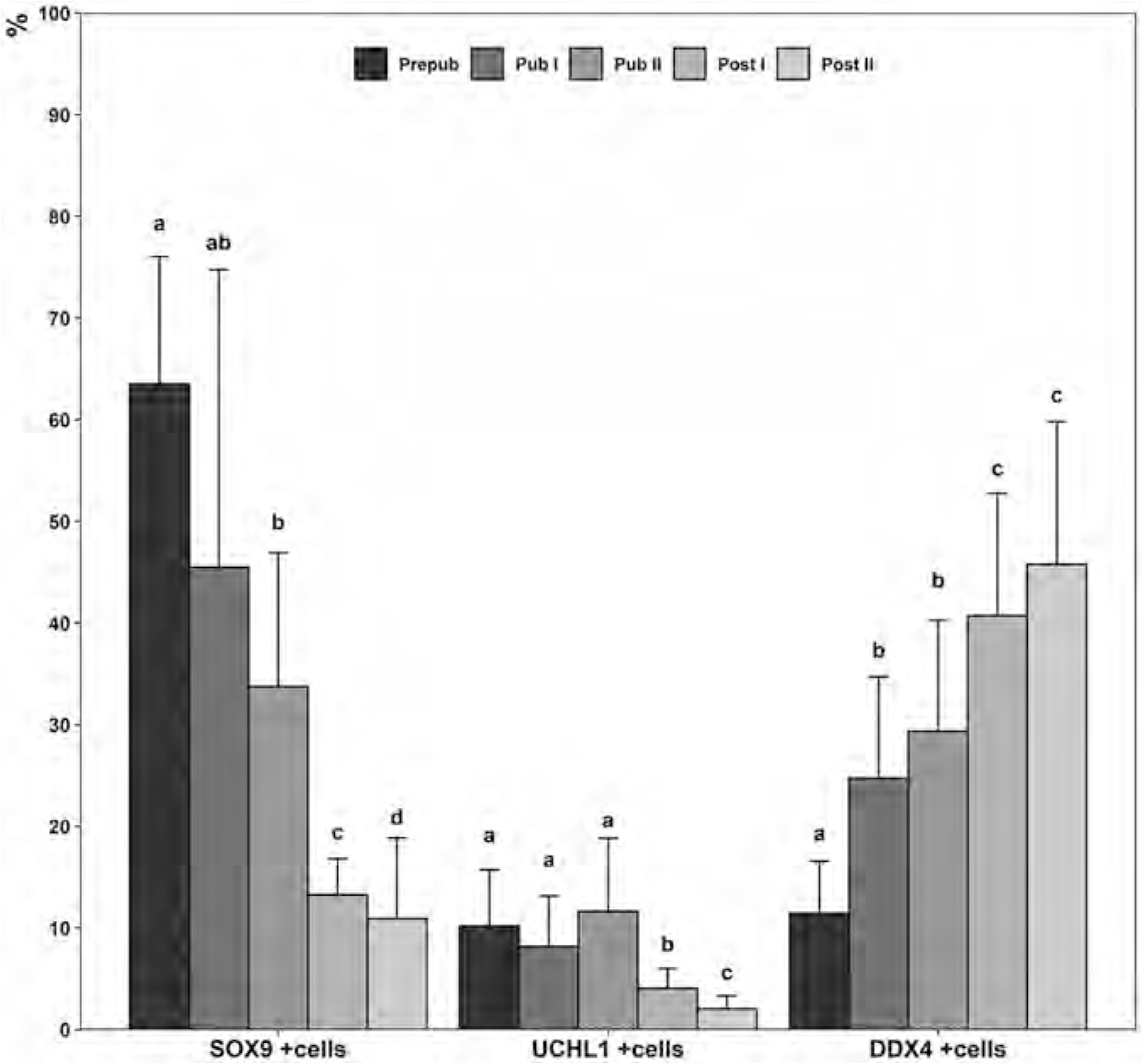
Percentages of SOX9, UCHL1 and DDX4 positive cells in relation to all DAPI positive cells per seminiferous tubule cross-section at different developmental phases of domestic cats. Data of 24 different seminiferous tubule cross-sections from two animals per developmental phase are displayed as means ± SD. Significant differences between developmental phases are indicated by different letters (P < 0.05).

Quantification of SOX9+ nuclei per seminiferous tubule cross-section revealed no changes in their abundance between developmental phases (Fig. 4 A). However, the proportional representation of SOX9+ nuclei decreased significantly from about 62 % in the prepubertal phase to 9 % in the postpubertal II phase as the number of germ cells increased by spermatogenetic activity (Fig. 5).

Average numbers of UCHL1+ spermatogonia per seminiferous tubule cross-section were significantly different between developmental phases, with the lowest found in the prepubertal phase (Fig. 4 B), yet their absolute number remained relatively stable after puberty began. Quantification of DDX4+ cells revealed that before puberty, total as well as relative numbers per tubule cross-section were similar to UCHL1+ spermatogonia (Fig. 4 C and Fig. 5). As puberty began, DDX4+ cell numbers increased significantly with progressing spermatogenesis, and reciprocally, the proportion of UCHL1+ spermatogonia continuously declined as more advanced spermatogenic cell types emerged and became more prominent.

### 3.7. Conserved labelling patterns for testicular cell markers in somatic and spermatogenic cells of wild feline species

**Fig. 6.**
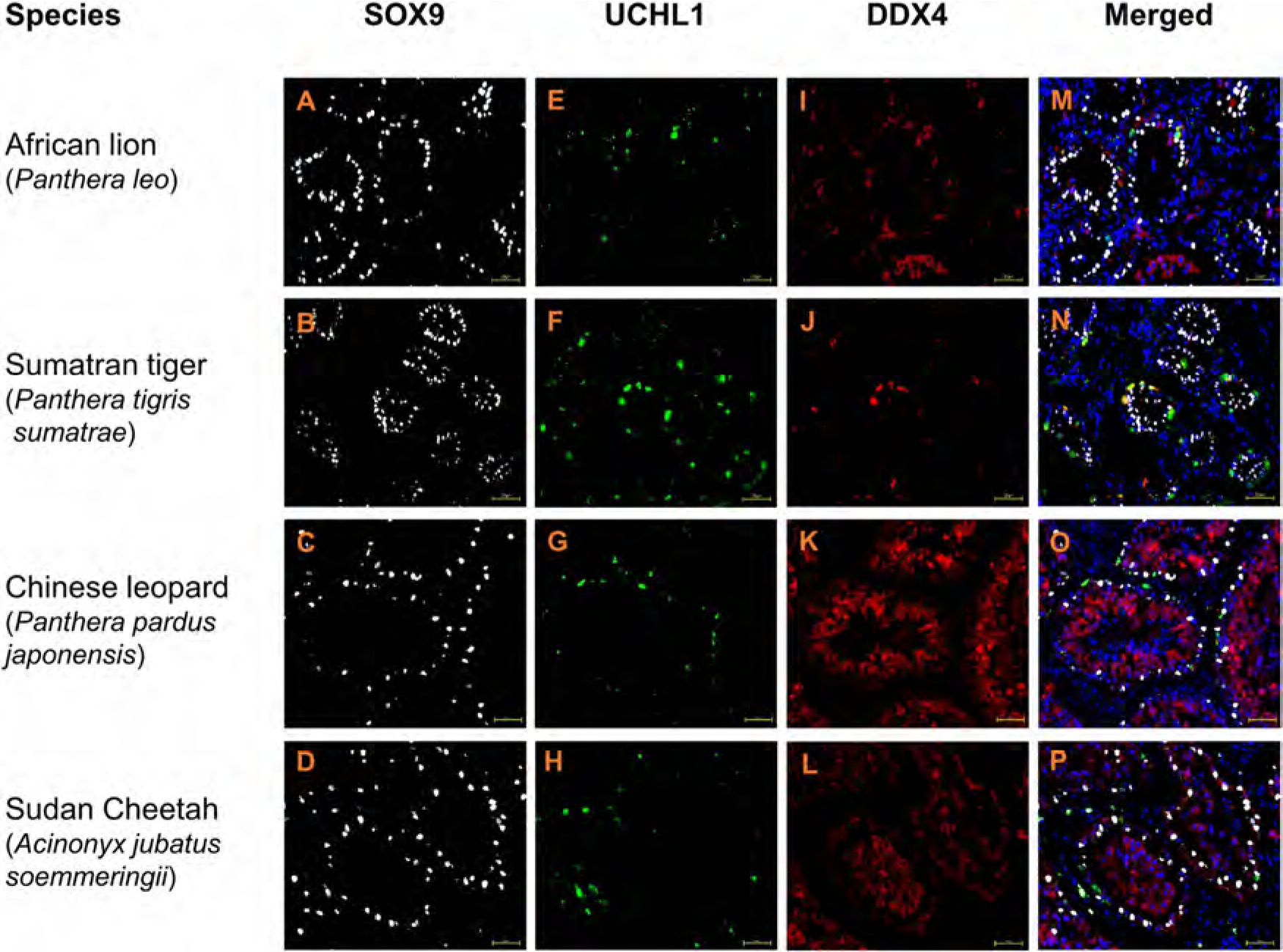
Cryo-embedded testis tissue of wild felids stained with SOX9 (white) UCHL1 (green) and DDX4 (red) antibodies. Nuclei (blue) stained with DAPI. Scale bar: 50 μm.

**Fig. 7.**
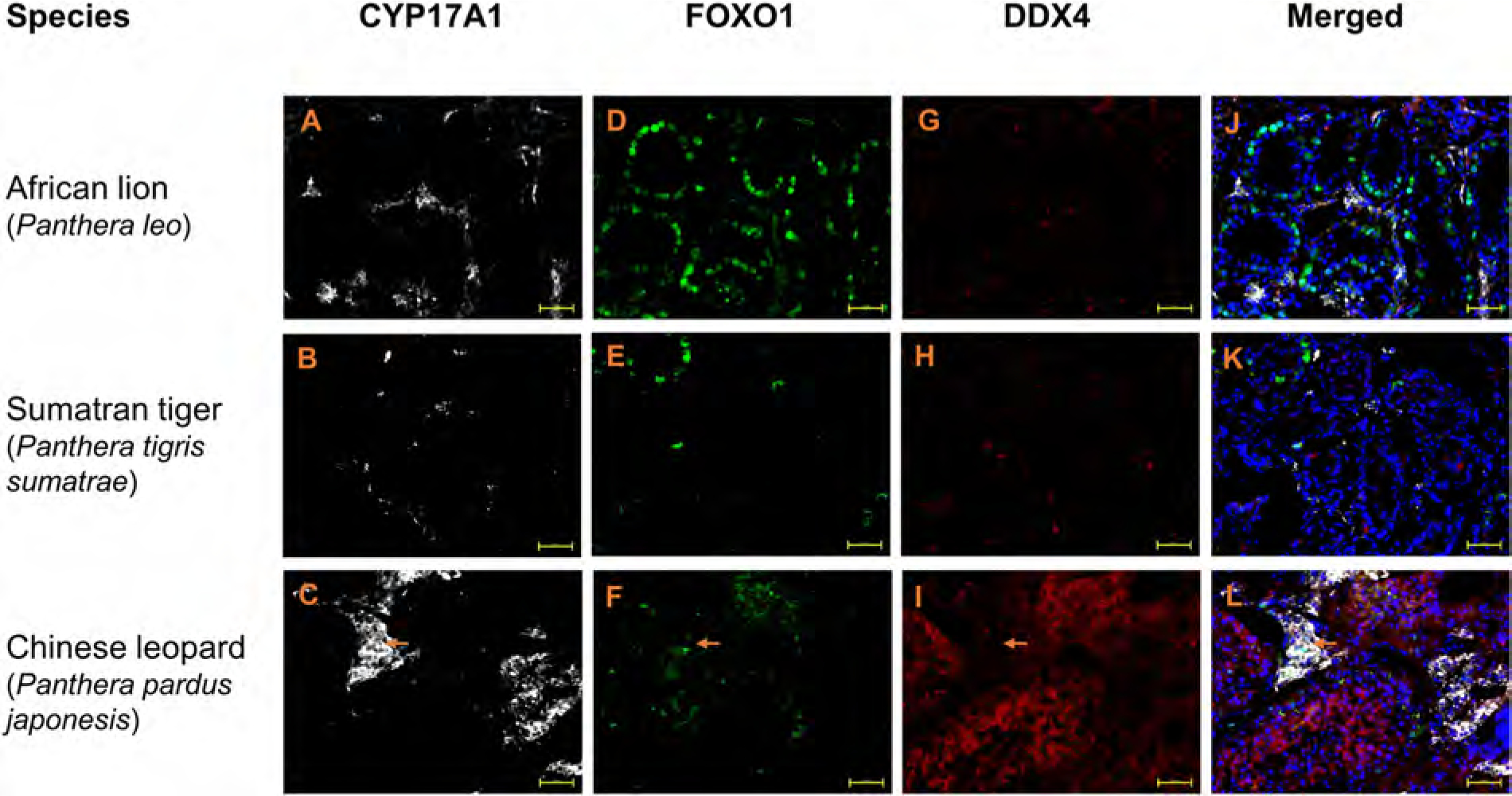
Cryo-embedded testis tissue of wild felids stained with CYP17A1 (white), FOXO1 (green) and DDX4 (red) antibodies. Nuclei (blue) stained with DAPI. Scale bar: 50 μm. Note: Chinese leopard, an interstitial labelling was detected in the FOXO1 fluorescent channel (arrow). Staining with the corresponding secondary antibody in the absence of FOXO1 antibody showed also similar unspecific interstitial labelling (Suppl. Fig. 4).

In wild feline testes, SOX9 and CYP17A1 labelling was analogous to that observed in domestic cat testes. Specifically, nuclear SOX9+ labelling of Sertoli cells could be identified in seminiferous tubules of all wild species (Fig. 6 and Suppl. Fig. 3, A–D, respectively). Moreover, CYP17A1+ Leydig cells were evident in the interstitial compartment of testes from lion, Sumatran tiger and Chinese leopard (Fig. 7 A–C). For Sudan cheetah, we were not able to test CYP17A1 labelling due to insufficient testis fragments.

Cross-sections of testes from lion, Sumatran tiger, Chinese leopard and Sudan cheetah showed a spermatogonial UCHL1 labelling pattern similar to that of domestic cat (Fig. 6 E–H and Fig. 1 F–J). Likewise, spermatogonial FOXO1 labelling was seen in lion and Sumatran tiger testes (Fig. 7 D and E), whereas in cross sections of Chinese leopard testes, additional background interstitial fluorescence signal was detected (Fig. 7 F) which was similar in negative controls (Suppl. Fig. 4 H). DDX4 was broadly detected in germ cells of testes from all wild felids (Fig. 6 I–L and Fig. 7 G–I).

## 4. Discussion

As the need to conserve wild felid species grows, so do the opportunities to apply advanced assisted reproductive technologies towards the goal of preserving these valuable species. However, it is difficult and unethical to prospectively sample the previous testicular tissue of endangered wild felid species. In their place, the domestic cat provides the ready opportunity to evaluate testes across postnatal development, benchmark marker expression and establish robust preservation strategies that can later be applied to endangered wild felids. The present study sought to immunolocalize somatic and spermatogenic cell types in the testes of domestic cats using antibodies directed towards five selected marker proteins and correlate these results with four different wild feline species. Our results demonstrate a largely expected pattern or marker labelling in domestic cats based on prior results in other mammalian species and a high concordance in marker labelling in corresponding wild felids.

Spermatogenesis is well studied in laboratory rodents and primates (including humans), but knowledge about felid spermatogenesis is relatively limited. Nevertheless, among species that have been examined, the identity of spermatogenic cell types, organisation and timing of their development, and many of the underlying molecular and cellular processes involved in spermatogenesis appear to be highly conserved. As a first step towards characterising testicular cell types in felids, we looked for markers in fixed tissue sections and found that wild feline species exhibit the same degree of conservation of labelling for several markers and identity of the spermatogenic cell types observed in the domestic cat testis.

At all of the feline testicular developmental phases examined and in all of the species examined, Sertoli and Leydig cells, two major testicular somatic cell types, were identified based on labelling for SOX9 and CYP17A1, respectively. During foetal testis development, the transcription factor SOX9 plays a major role in gonadal sex determination [25]. SOX9 expression is activated by SRY in precursors of foetal Sertoli cells by 13.5 days post coitum in mice [26] and is sustained throughout the life of Sertoli cells [27]. The present study demonstrated for the first time nuclear SOX9 staining in feline Sertoli cells of domestic cat, lion, Sumatran tiger, Chinese leopard and Sudan cheetah.

We found that the average number of SOX9-positive cells per seminiferous tubule cross-section did not change between developmental phases. In postnatal mice, Sertoli cell numbers per testis increase 6-fold between day 1 and day 20, but subsequently remain unchanged [28], including across the cycle of the seminiferous epithelium in adults [29]. But domestic cats are moderate seasonal breeders and undergo cyclic regression and regeneration of the seminiferous epithelium [23,30]. In another seasonal breeding species like the roe deer, seasonal regression and regeneration of spermatogenesis only occurs among spermatogenic cells, while Sertoli cell numbers per tubule do not change with season [31]. Comparable to this, we assume that a constant number of Sertoli cells might also be a characteristic of male domestic cats whose testes were not only collected at different developmental phases but also at variable times of the year.

CYP17A1 is involved in the biosynthesis of testosterone and catalysers the 17-α-hydroxylation and the 17,20-lyase reaction for the conversion of pregnenolone and progesterone to dehydroepiandrosterone and androstenedione, respectively [32,33]. Detection of transcripts encoding CYP17A1 is possible by quantitative real-time PCR in testes of foetal mice by 12.5 days post coitum [26], foetal human testes at 8–10 gestational weeks [34] and foetal testis of domestic cat at day 34 of pregnancy and later [35]. Confirming our prior observations [20], robust labelling for the CYP17A1 steroidogenic enzyme was detected on interstitial Leydig cells and found at all development phases of postnatal domestic cat testes. However, improving upon the prior observations, the antibody used in the present study was specific to interstitial Leydig cells and did not label spermatids. Moreover, we found similar CYP17A1 labelling of Leydig cells in the lion, Sumatran tiger and Chinese leopard. In conclusion, the main somatic cell types can be unequivocally identified in testes of male felids by nuclear SOX9 in Sertoli and cytoplasmic CYP17A1 in Leydig cells.

Spermatogenic cell types are more difficult to distinguish because markers of a given cell type do not disappear suddenly during transition to the next germ cell stage. Therefore, marker combinations are commonly used to classify germ line cells as for instance spermatogonia which are hardly to distinguish by morphological criteria. One such marker is UCHL1, a deubiquitinating enzyme [36,37] predominantly expressed in the brain where it can comprise up to 5 % of total neuronal proteins [38]. UCHL1 is also expressed in gonads of various species [39], but the testicular expression pattern appears to vary between species. For instance, mouse UCHL1 is detectable in both spermatogonia and Sertoli cells [40], while spermatogonia-specific expression had been observed in bovine [41–43], ovine [44,45], porcine [46,47], primate [48] and feline species, including domestic cat, cheetah (*Acinonyx jubatus*) and Amur leopard (*Panthera pardus orientalis*) [19]. In contrast, another study observed UCHL1 labelling not only in spermatogonia but also in early spermatocytes of testes from adult domestic cats [17]. The most likely explanation for these divergent results is the use of different antibodies. Our findings demonstrated that the anti-UCHL1 antibody labelled spermatogonia of domestic cat, lion, Sumatran tiger, Chinese leopard and Sudan cheetah. The exception to this specificity was apparent interstitial staining in individual postpubertal samples of domestic cat. In one previous study, Choi et al. [49] reported a phase-dependent expression of UCHL1 in spermatogonia, Leydig cells and myoid cells of donkey testes. Although we are unable to exclude the possibility of autofluorescence and/or secondary antibody labelling in the interstitial compartments of the cryo-embedded control samples in the absence of UCHL1 antibody, immunostaining of paraffin-embedded testis fragments revealed an interstitial labelling with UCHL1 antibody in only one domestic cat individual at the postpubertal II phase. An increased level of cellular autofluorescence may arise from the accumulation of the autofluorescent pigment, lipofuscin as shown in Leydig cells isolated from chronically stressed or aged rats [50] or in senescent or chronically stressed human fibroblasts [50,51]. Therefore, detection of lipofuscin levels in feline testes at postpubertal phase may reveal information underlying the interstitial autofluorescence in adult individuals.

Previous studies posited that UCHL1 is abundant in both undifferentiated and differentiating spermatogonia of prepubertal and adult domestic cats [17,19]. This was based on partially overlapping labelling with markers of undifferentiated spermatogonia such as ZBTB16 (Zinc finger and BTB domain containing protein 16) and POU5F1 (POU domain, class 5, transcription factor 1) that were already shown being expressed in cat testes at the mRNA level [52]. Since colocalisation of all three markers in a subset of spermatogonia supports expression in undifferentiated spermatogonia [19], the portion of UCHL1 positive spermatogonia that do not express undifferentiated spermatogonia markers would presumably comprise differentiating spermatogonia. Our observation of increasing UCHL1+ spermatogonial numbers during the pubertal phase when differentiating spermatogonia emerge supports this assumption.

Separately, FOXO1 is required for SSC homoeostasis and it serves as undifferentiated spermatogonia marker in the mouse [53]. FOXO1 belongs to the winged helix/ forkhead transcription factors family [54] whose members play major roles in several cellular processes including cell cycle arrest, repair of damaged DNA and apoptosis [55]. Previously, FOXO1 was detected in a small spermatogonia population of domestic cat by Tarnawa et al. [56] and was detected with the same antibody in the cytoplasm of prospermatogonia and spermatogonia of domestic cats at immature and adult phases [17]. Labelling of serial sections was used to determine whether FOXO1 and UCHL1 were coexpressed and ~90% overlap was reported [17]. Here, we confirmed this with direct co-labelling and found that the vast majority of spermatogonia expressed both markers. Importantly, FOXO1 staining in the spermatogonia of domestic cat was observed across developmental phases and in wild species (lion and Sumatran tiger). In the present study, distinct subcellular labelling patterns for FOXO1 were detected in spermatogonia. Before and during puberty, cytoplasmic localisation dominates, which points to a likely role in regulating spermatogenesis given knowledge that cytoplasmic-nuclear translocation regulates FOXO1 function. Translocation of FOXO1 from the nucleus to the cytoplasm is associated with the transition of prospermatogonia to spermatogonia and the reverse occurs during spermatogonial differentiation [53,57]. Whether the translocation of FOXO1 in feline spermatogonia occurs in a similar way and bears the same biological importance as in the murine model remains to be proven. Within this context, additional analyses would be required to investigate the physiological conditions of spermatogonia that trigger FOXO1 expression and/or changes to its localisation pattern.

The cytoplasmic RNA helicase DDX4 (also known as VASA) belongs to the Dead-box proteins family [58] and exhibits a highly-conserved germline expression profile from flies [59] to mice [60], rats [61], marsupials and monotremes [62], rhesus macaques [63] and humans [64]. As expected and in contrast to the spermatogonia-specific markers UCHL1 and FOXO1, DDX4 was detected in a wide range of feline germ cells from spermatogonia to round spermatids. In cats, the literature on DDX4 labelling of germ cells is highly variable, from labelling restricted to differentiated germ cells [17] or in both undifferentiated and differentiated germ cells [65], to only detectable with paraffin-embedded testis tissue [21], or absent entirely when cryo-embedded testis tissue was employed [21]. Our results resolve these conflicts by demonstrating broad labelling in germ cells of cryo-embedded testis fragments from domestic cat, lion, Sumatran Tiger, Chinese leopard and Sudan cheetah. In addition to our developmental phase classification by ploidy analysis, the quantification of tubular DDX4 positive cells therefore seems to be suitable to clearly distinguish prepubertal, pubertal and postpubertal individuals. Finally, DDX4 can be used to confirm the germ cell lineage of testicular cells at different developmental phases since no somatic cell types were labelled by this antibody.

In conclusion, the specific characterisation of different types of domestic cat testicular cells was possible using a set of antibodies that were earlier applied in spermatogenesis studies in different mammal species. At all developmental phases, the two major somatic cell types of the testis, Sertoli and Leydig cells, were identified by SOX9 and CYP17A1 labelling, respectively. Spermatogonia are positive for UCHL1 and/or FOXO1, whereas, DDX4 was restricted to spermatogenic cells from spermatogonia through round spermatids. Overall, conserved immunolocalization patterns in testes of four different wild feline species demonstrated the validity of using the domestic cat as a model for endangered feline species. Future studies that identify cell surface selectable markers to permit enrichment of particular spermatogenic cells could use the markers investigated here to confirm cell selection in isolated cells. For instance, validated populations of undifferentiated spermatogonia selected in this way could be used for *in vitro* culture to expand cells for transplantation to restore spermatogenesis *in vivo*, derive more advanced germ cells through *in vitro* spermatogenesis, or be used for basic biological investigation of their features such as metabolism, signalling and development. Ultimately, the ability to definitively identify testicular cell types in complex mixtures or validate their purity after enrichment, as demonstrated here, will be central to the success of advanced investigation to use those cells for therapeutic purposes.

## Supplementary Material

Appendix A. Supplementary material 1

Appendix A. Supplementary material 2

## Figures and Tables

**Table 1:** Primary antibodies used for the characterisation of feline testicular cells.

Antigen	Host-antibody type	Manufacturer	Catalogue number	Working dilution cryo	Working dilution paraffin

DDX4	Goat-polyclonal IgG	R&D systems	AF2030	1:40	-
SOX9	Rabbit-monoclonal IgG	Abcam	ab185966	1:300	1:1.000
FOXO1	Rabbit-monoclonal IgG	Cell signal	2880	1:50	-
UCHL1	Mouse-monoclonal IgG	Abcam	ab8189	1:500	1:1.000
CYP17A1	Mouse-monoclonal IgG	Santa Cruz Biotechnology	sc374244	1:4000	-

**Table 2:** Secondary antibodies used for the characterisation of feline testicular cells.

Secondary antibody	Host-antibody type	Conjugate	Catalogue number	Working dilution

Anti-goat	Donkey-polyclonal IgG	Alexa Fluor^®^ 647	A-21447	1:200
Anti-rabbit	Donkey-polyclonal IgG	Alexa Fluor^®^ 568	A-10042	1:200
Anti-goat	Donkey-polyclonal IgG	Alexa Fluor^®^ 568	A-11057	1:200
Anti-mouse	Donkey-polyclonal IgG	Alexa Fluor^®^ 568	A-10037	1:200
Anti-mouse	Donkey-polyclonal IgG	Alexa Fluor^®^ 488	A-21202	1:200
Anti-rabbit	Donkey-polyclonal IgG	Alexa Fluor^®^ 488	A-21206	1:200
Anti-mouse	Horse-polyclonal IgG	POD	MP-6402	ready to use
Anti-rabbit	Horse-polyclonal IgG	POD	MP-6401	ready to use

**Table 3:** Classification of developmental phases in domestic cats based on testis weight and flow cytometric ploidy analysis.

Developmental phase	Individual	Testis weight [mg]	1C/4C	1C/2C	2C/4C	Completion of spermatogenesis

Prepubertal	1	136	0.48	0.01	29.2	-
	2	140	0.02	0.002	18.80	-
Pubertal I	1	380	0.07	0. 02	3.33	-
	2	398	0.20	0.05	4.24	-
Pubertal II	1	519	0.27	0.11	2.53	-
	2	596	0.53	0.19	2.74	-
Postpubertal I	1	917	1.18	0.98	1.21	+
	2	1100	2.75	0.96	2.87	+
Postpubertal II	1	1204	3.61	2.12	1.7	+
	2	1975	4.5	4.05	1.11	+

1C, haploid; 2C, diploid; and 4C, tetraploid cells.
