# Supplementary material for: Molecular phenotyping of domestic cat (Felis catus) testicular cells across postnatal development – A model for wild felids
Source: Theriogenology Wild. Author manuscript; Available in PMC 2023 Jul 17. (PMC10350788; doi:10.1016/j.therwi.2023.100031)
Supplement: Appendix A. Supplementary material 1 [file NIHMS1912220-supplement-Appendix_A__Supplementary_material_1.ppt]

## Slide 1
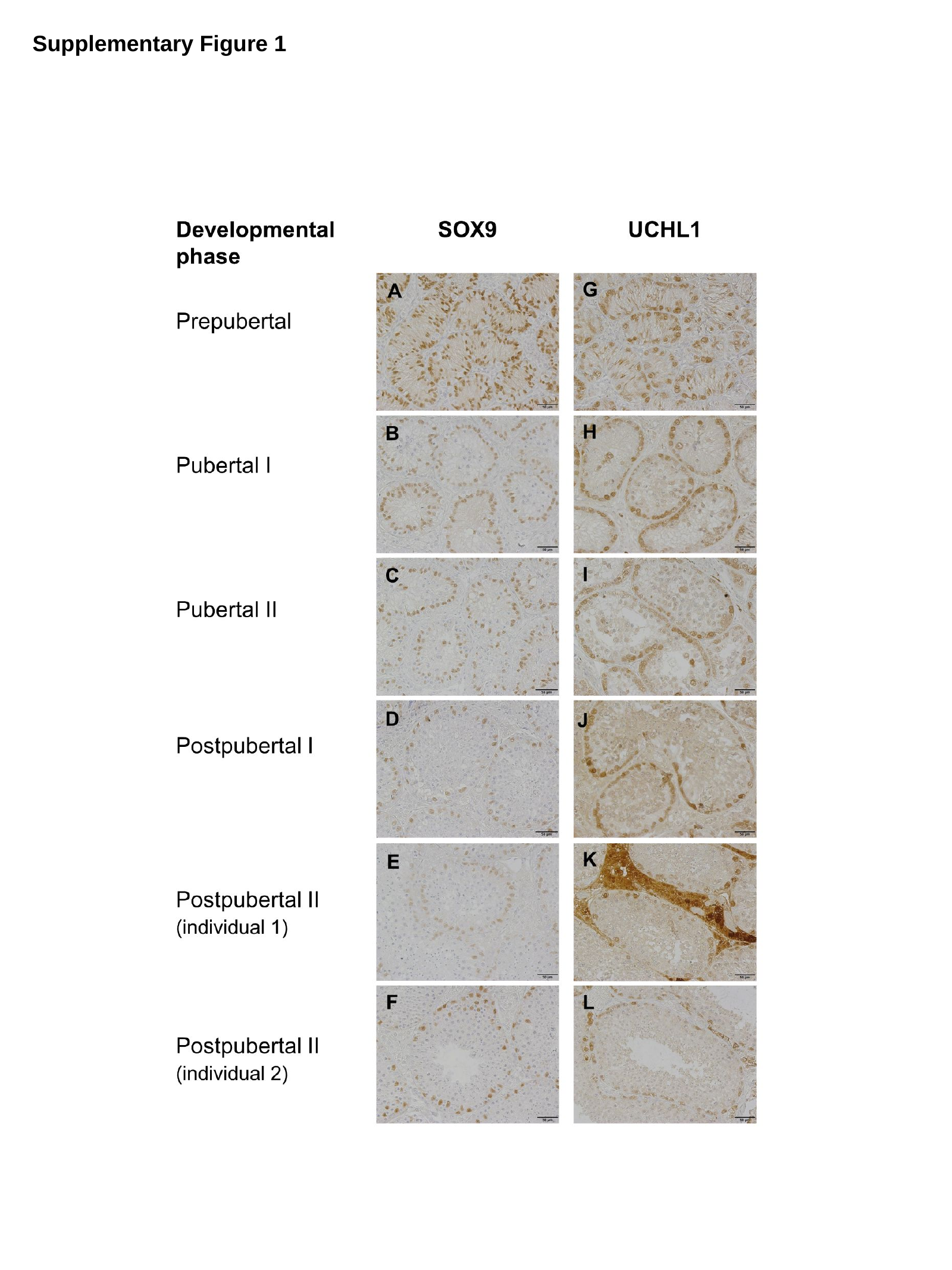

Supplementary Figure 1

## Slide 2
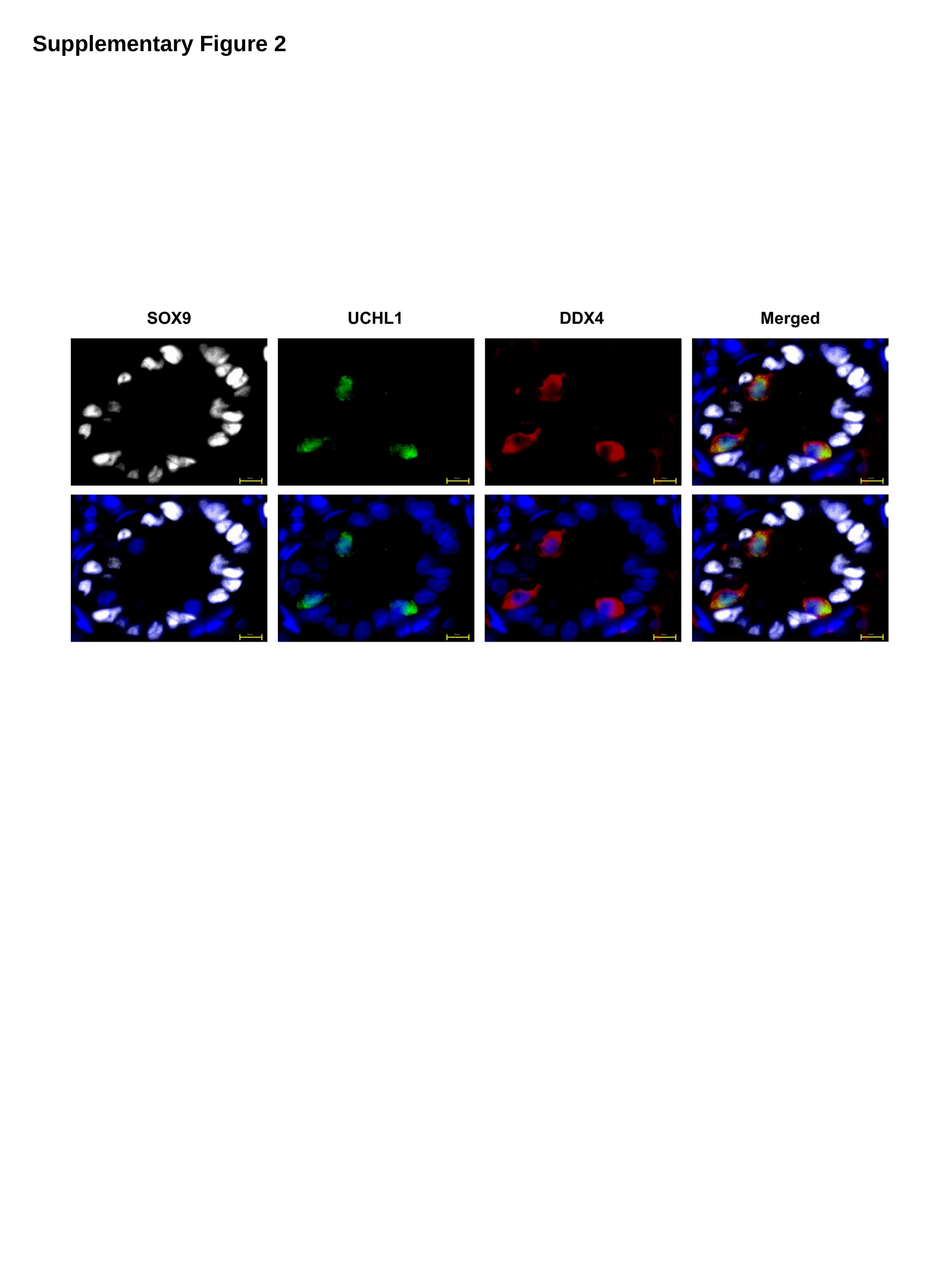

Supplementary Figure 2

## Slide 3
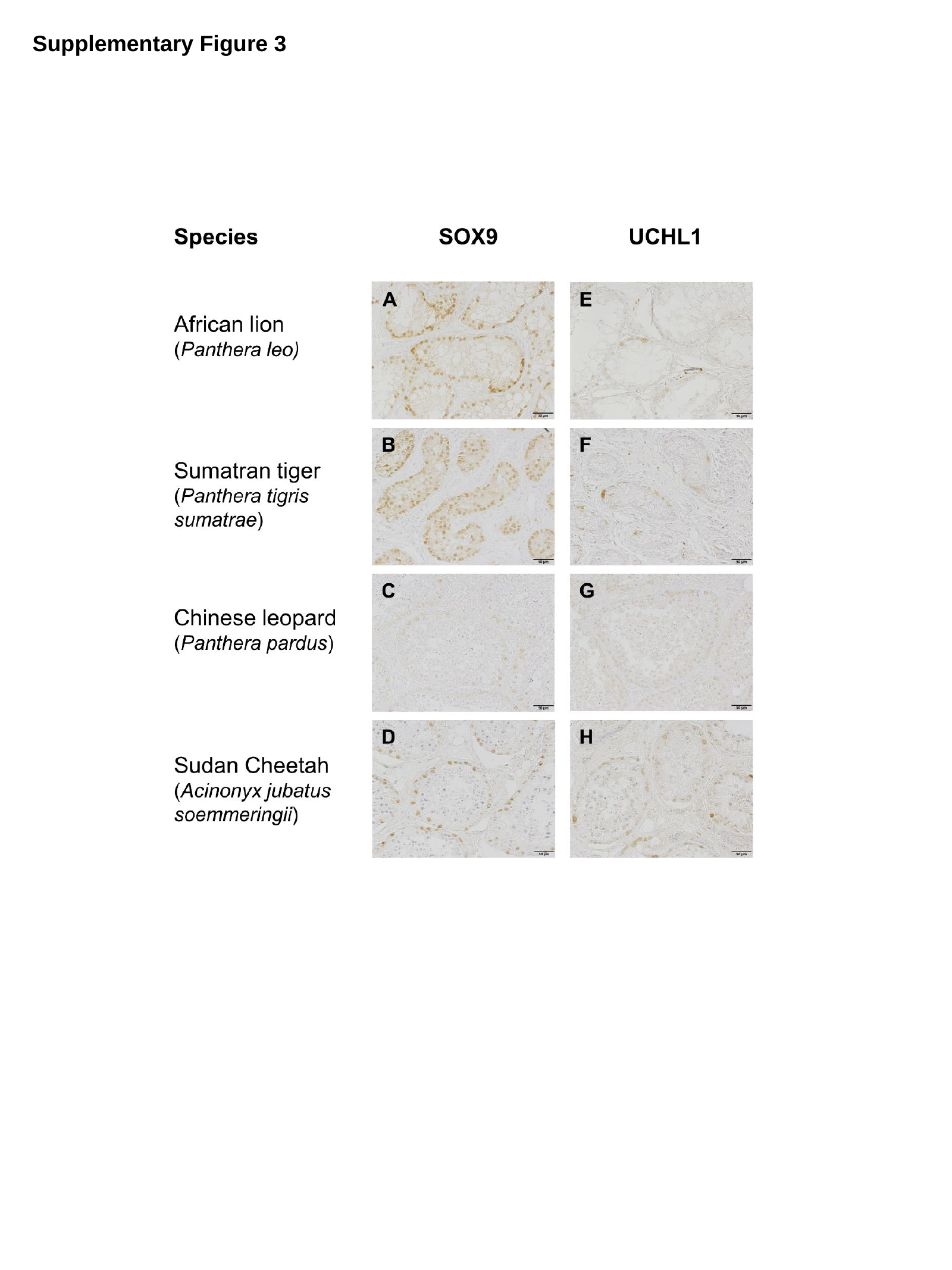

Supplementary Figure 3

## Slide 4
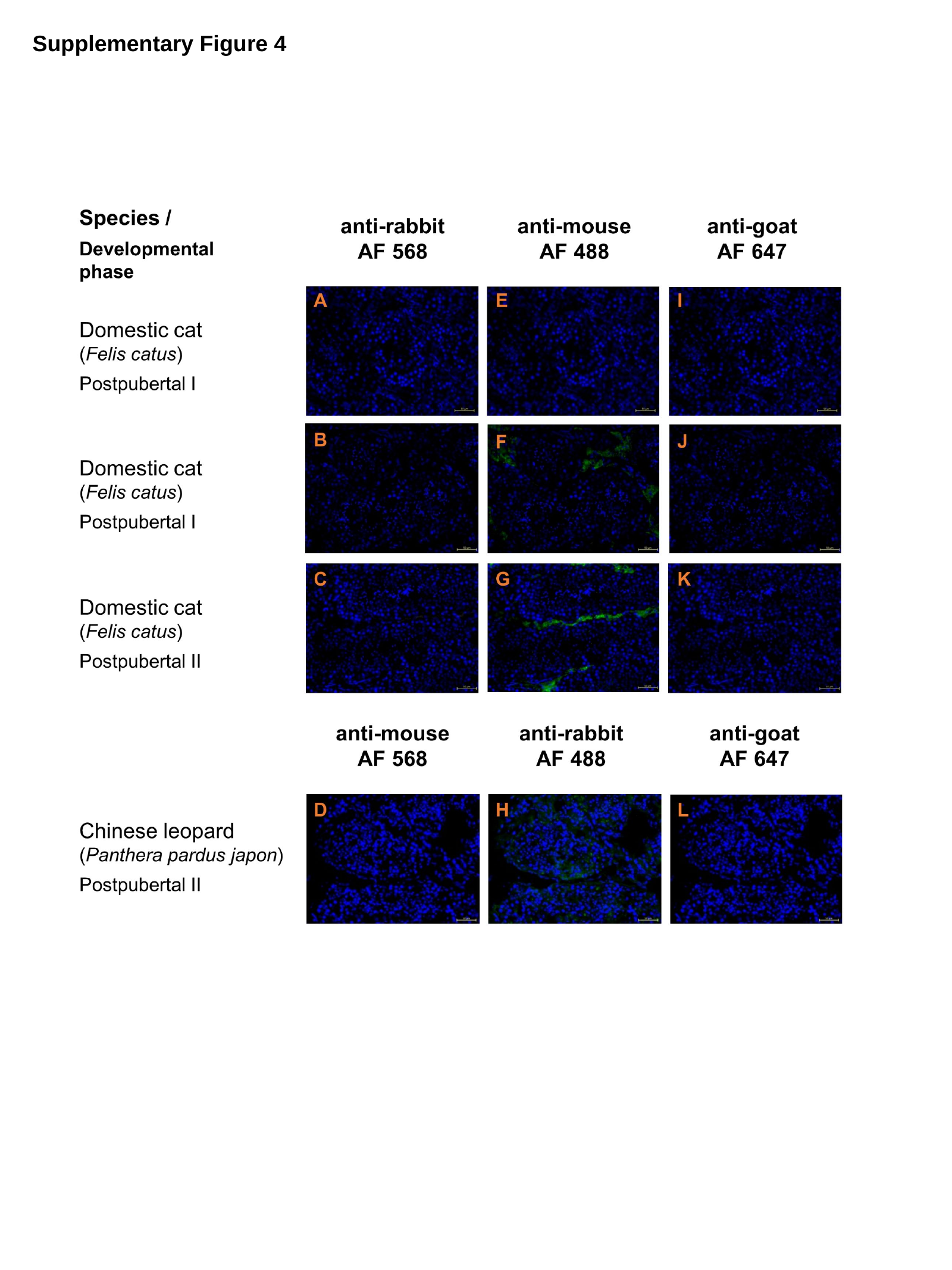

Supplementary Figure 4

## Slide 5
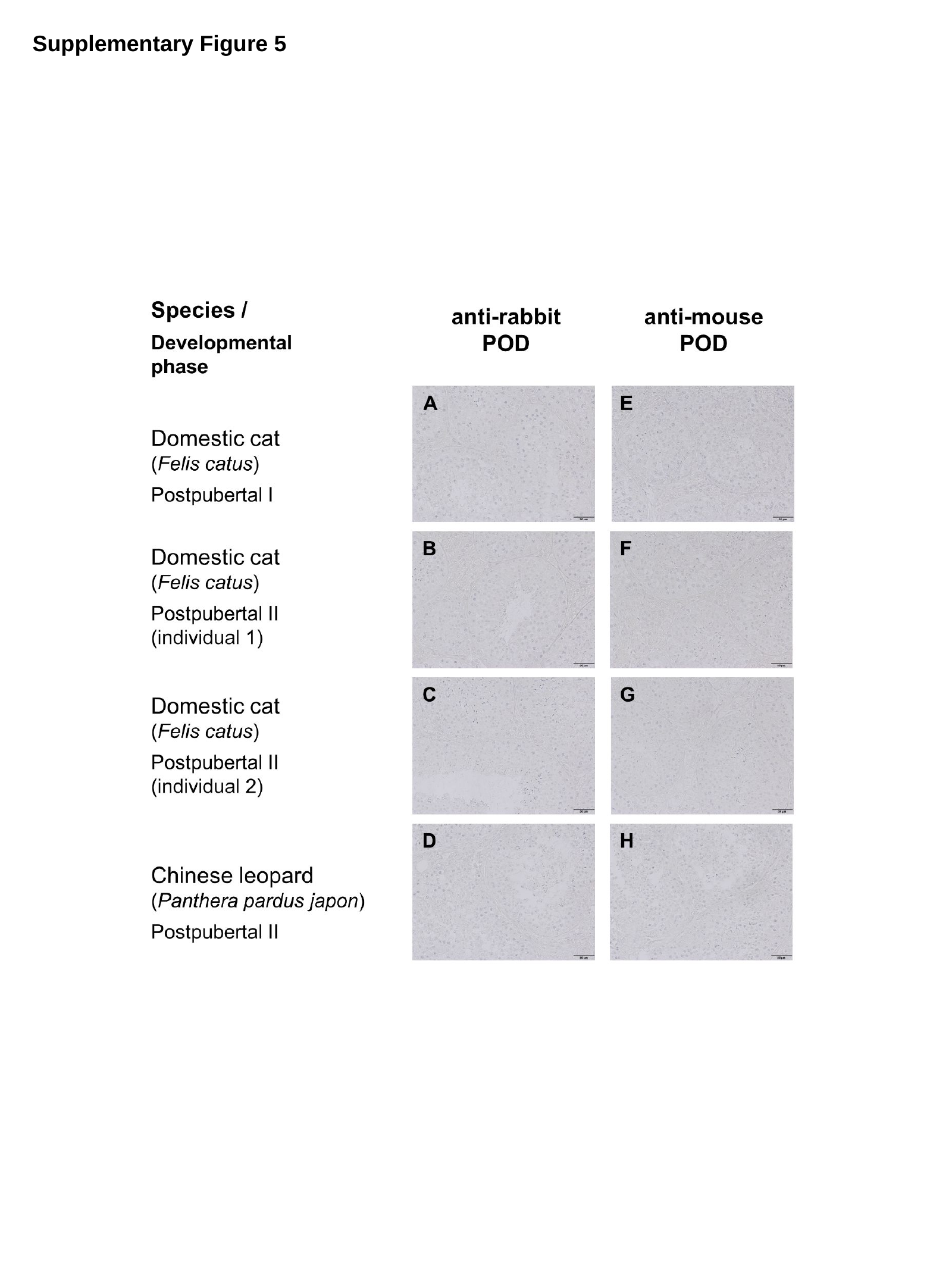

Supplementary Figure 5
