# Supplementary material for: Molecular phenotyping of domestic cat (Felis catus) testicular cells across postnatal development – A model for wild felids
Source: Theriogenology Wild. Author manuscript; Available in PMC 2023 Jul 17. (PMC10350788; doi:10.1016/j.therwi.2023.100031)
Supplement: Appendix A. Supplementary material 2 [file NIHMS1912220-supplement-Appendix_A__Supplementary_material_2.docx]

**Supplementary Table 1** Classification of developmental phases in four wild feline species based on flow cytometric ploidy phase analysis.

| **Species / Age**  (years, months) | **n** | **Testis weight**  [g] | **1C/4C** | **1C/2C** | **2C/4C** | **Developmental phase /**  **completion of spermatogenesis** |
| --- | --- | --- | --- | --- | --- | --- |
| African lion (1,6) | 1 | 5.77 | 0.11 | 0 | 26.45 | Prepubertal (-) |
| Sumatran tiger (1,5) | 1 | 3.44 | 0.28 | 0.008 | 35.79 | Prepubertal (-) |
| Chinese leopard (13,9) | 1 | 10.34 | 5.26 | 2.04 | 2.58 | Postpubertal II (+) |
| Sudan cheetah (15,11) | 1 | 4.42 | 2.22 | 1.52 | 1.45 | Postpubertal II (+) |

1C, haploid; 2C, diploid; and 4C, tetraploid cells.

Completion of spermatogenesis was determined by the presence (+) of spermatozoa in the cauda epididymis. A decrease in ratio 2C/4C indicates an increase in cell division (mitosis and first part of meiosis); an increase in ratio 1C/2C indicates an increase in meiotic activity, and thus, sperm production.

**Supplementary Figure 1** Paraffin-embedded testis tissue of domestic cats from five developmental phases stained with SOX9 or UCHL1 antibodies. Scale bar: 50 µm. Note: At postpubertal II phase, an interstitial labelling with UCHL1 antibody was detected in one male (K) whereas the second male (L) showed no interstitial labelling. No staining was observed with the corresponding secondary antibody (anti-mouse POD) in the absence of UCHL1 antibody (Suppl. Fig. 5).

**Supplementary Figure 2** Cryo-embedded testis tissue of the domestic cat from prepubertal phase stained with SOX9 (white), UCHL1 (green) and DDX4 (red) antibodies. Nuclei (blue) stained with DAPI. Scale bar; 10 µm. Magnification 100X.

**Supplementary Figure 3** Paraffin-embedded testis tissue of wild felids stained with SOX9 or UCHL1 antibodies. Scale bar: 50 µm. Note: In the African lion (E) and Sumatran tiger (F), a dot-like interstitial labelling with UCHL1 antibody was detected. The dot-like labelling might be related to precipitated antibody molecules. No staining was observed with the corresponding secondary antibody (anti-mouse POD) in the absence of UCHL1 antibody (Suppl. Fig. 5).

**Supplementary Figure 4** Selected control samples of cryo-embedded testis tissue of domestic cats and wild felids only exposed to secondary antibodies. Primary antibodies were omitted. Note that interstitial labelling was detected in few individual samples for the Donkey anti-mouse AF 488 (F, G) and Donkey anti-rabbit AF 488 (H) secondary antibody. The dot-like labelling (H) might be related to precipitated antibody molecules. All other controls did not reveal unspecific labelling as exemplary shown in this figure (A-E, I-L). Nuclei (blue) stained with DAPI. Scale bar: 50 µm. AF, Alexa Fluor.

**Supplementary Figure 5** Selected control samples of paraffin-embedded testis tissue of domestic cats and wild felids only exposed to secondary antibodies. Primary antibodies were omitted. All controls did not reveal unspecific labelling as exemplarily shown in this figure (A-D, E-H). Scale bar: 50 µm.
